# Ablation of insulin-producing cells prevents obesity but not premature mortality caused by a high-sugar diet in *Drosophila*

**DOI:** 10.1098/rspb.2014.1720

**Published:** 2015-02-07

**Authors:** Sara Naif Al Saud, Adam C. Summerfield, Nazif Alic

**Affiliations:** Institute of Healthy Ageing, and Research Department of Genetics, Evolution and Environment, University College London, Darwin Building, Gower St., London WC1E 6BT, UK

**Keywords:** ageing, *Drosophila melanogaster*, high-sugar diet, obesity, insulin/IGF-like signalling

## Abstract

Ageing can be modulated by genetic as well as nutritional interventions. In female *Drosophila melanogaster*, lifespan is maximized at intermediate concentrations of sucrose as the carbohydrate source, and yeast as the protein source. Dampening the signal through the insulin/IGF signalling (IIS) pathway, by genetic ablation of median neurosecretory cells (mNSCs) that produce insulin-like peptides, extends lifespan and counteracts the detrimental effects of excess yeast. However, how IIS reduction impacts health on a high-sugar diet remains unclear. We find that, while the ablation of the mNSCs can extend lifespan and delay the age-related decline in the health of the neuromuscular system irrespective of the amount of dietary sugar, it cannot rescue the lifespan-shortening effects of excess sugar. On the other hand, ablation of mNSCs can prevent adult obesity resulting from excess sugar, and this effect appears independent from the canonical effector of IIS, *dfoxo*. Our study indicates that while treatments that reduce IIS have anti-ageing effects irrespective of dietary sugar, additional interventions may be required to achieve full benefits in humans, where excessive sugar consumption is a growing problem. At the same time, pathways regulated by IIS may be suitable targets for treatment of obesity.

## 1. Introduction

Ageing is the major risk factor for a number of killer and debilitating diseases, including cancer, cardiovascular disease and neurodegeneration [1]. Together with an increasingly aged population, these non-communicable diseases are increasing in prevalence globally, posing a substantial burden on societies [2]. Recent work in biogerontology has revealed ageing as plastic, fuelling a drive towards direct treatments of ageing to replace the more conventional treatments for individual age-related diseases [1,3].

The lifespan of an organism is dependent on both extrinsic factors, such as nutrition, and intrinsic factors, such as the genetic makeup of an individual [3]. It has been known since 1935 that limiting the food intake of healthy rats can extend their lifespan [4]. This reduction in nutrient intake, called dietary or caloric restriction, can be implemented in different ways in a great number of model organisms, with the effect of extending their healthy lifespan [5]. In the females of the fruit fly *Drosophila melanogaster*, lifespan responds to both macronutrients in their diet: yeast as the source of protein and sucrose as the main carbohydrate. Lifespan is maximized at intermediate concentrations of each nutritional component [6,7]. However, the physiological responses to the two dietary components are different.

Female flies can draw immediate physiological benefits from excess yeast: increasing yeast consumption beyond the amount that maximizes lifespan will increase female fecundity [7,8], supporting the idea that protein dictates a trade-off between lifespan and fecundity, even though, in a mechanistic sense, this trade-off is not obligatory [9]. On the other hand, no benefit has been observed to arise from excess sugar: increasing sucrose in the diet actually reduces fecundity [7,8]. Furthermore, in adult flies, excess sugar leads to phenotypes similar to the human metabolic syndrome and its consequences. Flies fed on a high-sugar diet accumulate storage triacylglycerols (TAGs), becoming obese, develop insulin resistance and even heart disease [7,10,11]. In fruit flies, like in humans, the overconsumption of sugar appears to be toxic.

The network formed by the insulin/IGF signalling (IIS) and TOR (target of rapamycin) signalling pathways is an evolutionarily conserved modulator of lifespan [3]. Genetically dampening the signalling levels in the network can robustly extend the lifespan of a number of model organisms, and genes encoding the network components are associated with human longevity [3,12]. In *Drosophila*, IIS is initiated by one of seven *Drosophila* insulin-like peptides (*dilp*s) [13]. The genes encoding *dilp2*, *3* and *5* are expressed in the median neurosecretory cells (mNSCs) in the brain of the adult fly [14]. Partial ablation of these cells results in reduced *dilp* production and a robust extension of lifespan [14]. Interestingly, ablation of these cells can overcome the lifespan-shortening effects of increased yeast in the diet: the lifespan of the ablated flies is not reduced when yeast concentration is increased [15]. On the one hand, this indicates that mNSCs mediate the response of lifespan to yeast. On the other hand, it indicates that treatments based on dampening IIS in adults can overcome the effects of excess dietary protein.

It is currently unclear how the reduction in IIS, such as achieved by ablation of the mNSC, interacts with excess sugar in the diet. This question is important for two reasons. First, it would clarify whether changes in IIS also underlie the beneficial effects of reducing dietary sugar. Second, it would tell us whether interventions aimed at reduction of IIS in the adult can overcome the detrimental effects of excess dietary sugar. The latter aspect of this question is growing in importance, since the human consumption of sugar has tripled in the past 50 years [2]. This increase is one of the leading causes of obesity and the metabolic syndrome that plague the modern world, increasing the burden of chronic and age-related diseases [2,16]. The increase in sugar consumption is mostly owing to addition of sucrose, or its constituents, glucose and fructose, to numerous food products in the developed world [2]. With an increasing number of countries adopting a ‘western’ diet, the problem is becoming global [2]. *Drosophila* is increasingly used as a model to untangle the connections between dietary sugar, metabolism and health [17,18].

The experiments described herein examined whether reduction in IIS can counteract the negative effects of excess dietary sugar. With respect to mortality and age-related functional decline, we find that reduction in IIS achieved by the genetic ablation of mNSCs can be beneficial on both standard and high-sugar diets; however, it cannot protect specifically against the life-shortening effects of high sugar. On the other hand, downregulation of IIS can prevent the obese phenotype observed in adult female flies fed an excess of sugar, and this protection does not appear to depend on the canonical IIS pathway involving Forkhead Box O (FoxO) transcription factor (TF).

## 2. Material and methods

### (a) Fly husbandry and physiological assays

*dilp3GAL4*, *UAS-rpr* and the *dfoxo* mutant have been described [19]. All were backrossed at least six times into the wild-type outbred Dahomey population carrying the *w^1118^* mutation, and frequently outcrossed back into the same wild-type population. The Dahomey stock was collected in 1970 in Dahomey (now Benin) and has been kept in population cages maintaining its lifespan and fecundity at levels similar to freshly caught stocks. Combinations of transgenes/mutants were created using standard fly genetic techniques while avoiding population bottlenecks to generate the following (parental) fly lines: *w^1118^*/*w^1118^*; +/+; +/+, *w^1118^ UAS-rpr*/ *w^1118^ UAS-rpr*; +/+; +/+, *w^1118^*/*w^1118^*; +/+; *dilp3GAL4*/*dilp3GAL4*, *w^1118^*/*w^1118^*; +/+; *dfoxo*^Δ*94*^/TM6B, *w^1118^ UAS-rpr*/ *w^1118^ UAS-rpr*; +/+; *dfoxo*^Δ*94*^/TM6B, *w^1118^*/*w^1118^*; +/+; *dilp3GAL4 dfoxo*^Δ*94*^/TM6B.

The lines were maintained, and all experiments performed, at 25°C with 60% humidity and 12 L : 12 D cycle. Flies were maintained and experimental flies developed on sugar–yeast–agar (SYA) food (5% w/v sucrose, 10% w/v dried deactivated Brewers' yeast, 1.5% agar) [8].

Experimental flies were obtained by crossing suitable parental lines: in all experimental flies, transgenes were present in a single copy while homozygous *dfoxo* nulls were used. *UAS-rpr* and *dilp3GAL4* alone were used as controls for the ablation (*dilp3GAL4>rpr*) to account for any insertional mutagenic effects. Note that these two controls are necessary and sufficient to examine the phenotypes of the ablation.

The eggs were collected over 18 h and deposited into bottles containing SYA food at standardized densities. After emerging, flies were tipped into fresh SYA bottles, allowed to mate and females sorted on day two of adulthood onto food containing 5% w/v (1×) or 40% w/v (8×) sugar, 10% w/v dried deactivated Brewers' yeast, 1.5% agar. The mated females used in experiments were housed at 15 females per vial for climbing assays, 10 for all others. Separate cohorts of flies were used for the different assays. The flies were transferred to new food three times per week.

For lifespan assays, the survival of flies was assessed three times per week.

For climbing assays, climbing ability was scored once a week as follows. Flies were placed in a plastic 25-ml pipette (15 flies per pipette, three pipettes per condition), tapped to the bottom and allowed to climb for 45 s. Their position was then scored as low (climbing below 4 cm), medium (above 4 and below 22 cm) and high (above 22 cm). After two training trials, the scoring was repeated three times for each pipette and averaged to the nearest fly.

For TAG measurements, flies were frozen once a week or once a fortnight, as indicated in the relevant figure captions. At the end of the experiment, TAG was measured in batches where each batch contained a single fly from each time point/condition, by breaking individual flies in 400 µl 0.05% (v/v) Tween-20, inactivating the extracts at 70°C for 5 min, and measuring TAG against a commercial, standard TAG solution (ThermoScientific) using the Thermo Infinity TAG reagent according to the manufacturer's instructions (ThermoScientific). Around 10 measurements were made and the lowest and highest measurements removed from each time point/condition to guard against outliers.

### (b) Statistical analysis

**Figure 1.**
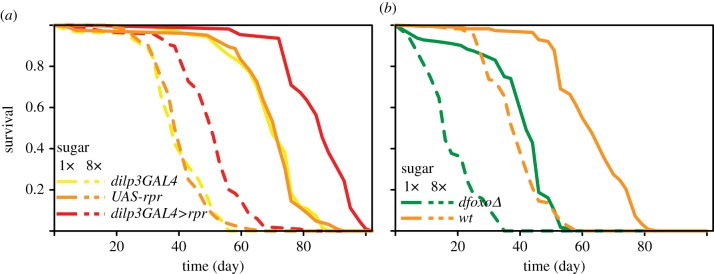
Ablation of mNSCs extends lifespan in the presence of excess sugar without alleviating the detrimental effect of the diet. (*a*) Lifespans of female flies ablated for the mNSCs (*dilp3GAL4>rpr*) or the two genetic controls (*dilp3GAL4* or *UAS-rpr* alone) on the food containing a healthy amount of sucrose (1×) or excess sucrose (8×). Statistical analysis of the data is shown in table 1. (*b*) Lifespans of wild-type or *dfoxo*Δ/*dfoxo*Δ female flies on food containing 1× or 8× sucrose. Statistical analysis of the data is shown in table 2.

Cox proportional hazards (CPH) analysis was performed using the *survival* package in R (http://CRAN.R-project.org/package=survival). When looking at the survival of the ablation on two sugar concentrations (figure 1*a* and table 1), the effects of transgenes were examined using two *a priori* contrasts: (i) ablation *versus* the controls, and (ii) *dilp3GAL4* control *versus UAS-rpr* control. The initial model included ‘8× sugar’ and ‘transgene’ as covariates and their interaction, and was then simplified by removing the non-significant interaction term. When examining the survival of *dfoxo* null flies on two sugar concentrations (figure 1*b* and table 2), the initial model included ‘8× sugar’ and ‘*dfoxoΔ*’ as covariates and their interaction, and was then simplified by removing the non-significant interaction term. When looking at the survival of the ablated flies in a wild-type and *dfoxo* null background (figure 3*c* and table 6), the model included ‘transgene’ and ‘*dfoxoΔ*’ as covariates and their interaction. Pairwise comparisons of survival were performed using the Log-rank test in JMP.

**Table 1.** Statistical analysis of data presented in figure 1*a*. CPH model with 700 dead and 29 censored events. The effects of transgenes present were assessed using two *a priori* contrasts: (i) ablation (*dilp3GAL4>rpr*) *versus dilpr3GAL4* and *UAS-rpr* alone controls, and (ii) *dilp3GAL4* control *versus UAS-rpr* control. Food was modelled as a categorical variable with 1× sugar as reference. The interaction between ‘transgene’ and ‘8× sugar’ was not significant and was removed from the final model. The coefficient estimate is the natural log of the hazard ratio where a negative value indicates a beneficial effect on survival.

coefficient	estimate	s.e.	*z*	*p*-value
transgene				
(i) ablation *versus* controls	−0.46	0.031	−15	< 2 ×10^−16^
(ii) *dilp3GAL3 versus UAS-rpr*	0.016	0.046	0.36	0.72
8× sugar	3.1	0.13	25	< 2 ×10^−16^

**Table 2.** Statistical analysis of data presented in figure 1*b*. CPH model with 458 dead and eight censored events. Both covariates were modelled as categorical variables with wild-type or 1× sugar as reference. The interaction between ‘*dfoxo*Δ’ and ‘8× sugar’ was not significant and was removed from the final model. The coefficient estimate is the natural log of the hazard ratio where a negative value indicates a beneficial effect on survival.

coefficient	estimate	s.e.	*z*	*p*-value
*dfoxo*Δ	2.4	0.14	17	< 2 ×10^−16^
8× sugar	2.6	0.14	19	<2 ×10^−16^

**Figure 2.**
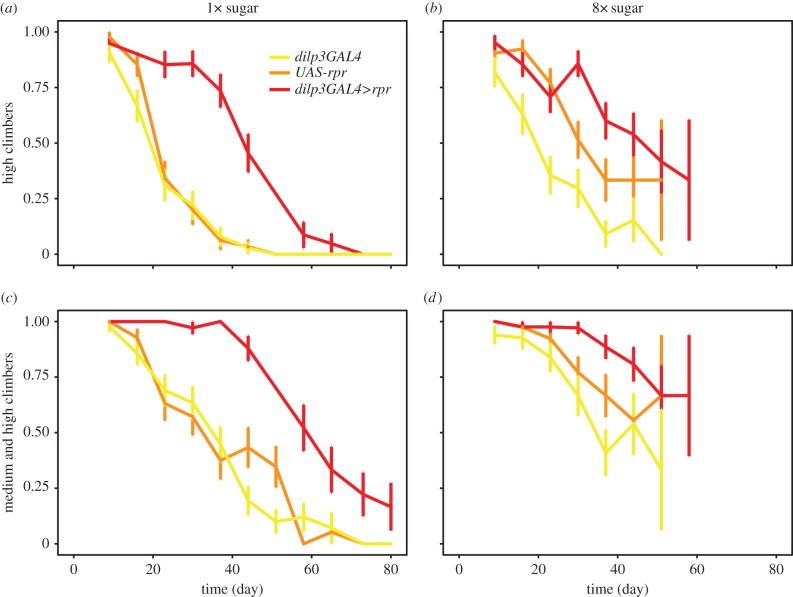
Beneficial effects of the mNSC ablation on climbing ability are observed irrespective of sugar content. Proportion of high climbers (climbing over 22 cm, (*a*,*b*)) or combined high and medium climbers (climbing over 4 cm, (*c*,*d*)) on the food containing a healthy amount of sucrose (1×, (*a*,*c*)) or excess sucrose (8×, (*b*,*d*)) in three cohorts of females flies with mNSC ablation (*dilp3GAL4>rpr*) or the two genetic controls (*dilp3GAL4* or *UAS-rpr* alone). The data from the three cohorts are combined, and the error bars show the standard error of the proportion. Statistical analysis of the data is shown in table 3.

Mixed effects ordinal logistic analysis was performed using the *ordinal* package in R (http://www.cran.r-project.org/package=ordinal) with the ‘fly cohort’ (vial) as the random effect (figure 2 and table 3). The effects of transgenes were again examined using two *a priori* contrasts: (i) ablation *versus* the controls, and (ii) *dilp3GAL4* control *versus UAS-rpr* control. The initial model included ‘age’, ‘transgene’, ‘8× sugar’, all of their pairwise interactions and the three-way interaction, and was simplified by sequentially removing non-significant interaction terms to give the final model.

**Table 3.** Statistical analysis of data presented in figure 2. Mixed effects ordinal logistic model with 1525 events and cohort (vial) as random effect. The effects of transgenes present were assessed using two *a priori* contrasts: (i) ablation (*dilp3GAL4>rpr*) *versus dilpr3GAL4* and *UAS-rpr* alone controls, and (ii) *dilp3GAL4* control *versus UAS-rpr* control. Food was modelled as a categorical variable with 1× sugar as reference. Colon (‘:’) indicates interaction term. The initial model included ‘age’, ‘transgene’ and ‘8× sugar’ as covariates, and all of their interactions, and was subsequently simplified by sequentially removing non-significant terms. The coefficient estimates are natural logs of odds of climbing high, where a negative value indicates a reduction in climbing ability.

coefficient	estimate	s.e.	*z*	*p*-value
age (day)	−0.12	5.9 ×10^−3^	−20	<2 ×10^−16^
8× sugar	−0.40	0.34	−1.2	0.22
transgene				
ablation *versus* controls	0.52	0.13	3.9	9.7 ×10^−5^
* dilp3GAL3 versus UAS-rpr*	0.24	0.20	1.2	0.23
age (day) : 8× sugar	0.025	0.010	2.5	0.012
age (day) : transgene				
ablation *versus* controls	9.6 ×10^−3^	3.1 ×10^−3^	3.1	1.9 ×10^−3^
* dilp3GAL3 versus UAS-rpr*	−3.0 ×10^−3^	6.1 ×10^−3^	−0.49	0.62
8× sugar : transgene				
ablation *versus* controls	−0.37	0.096	−3.9	1.1 ×10^−4^
* dilp3GAL3 versus UAS-rpr*	0.42	0.15	2.8	5.7 ×10^−3^

The performance index (electronic supplementary material, figure S1 and table S1) was calculated from the climbing counts data as ½(total number of flies observed + number of high climbers – number of low climbers)/total number of flies observed) as described [20]. The performance index data were analysed using a linear model in R. The initial model included ‘age’, ‘transgene’ and ‘8× sugar’ as covariates, all of their pairwise interactions and the three-way interaction, and was simplified by sequentially removing non-significant interaction terms and covariates to give the final model.

**Figure 3.**
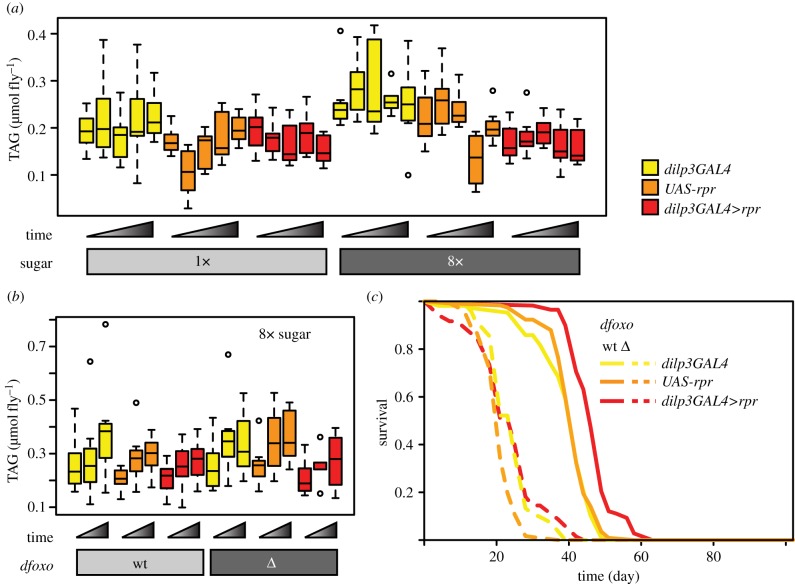
Ablation of the mNSCs prevents high-sugar-induced TAG accumulation independently of *dfoxo*. (*a*) Boxplots showing the levels of TAG measured in individual female flies with mNSC ablation (*dilp3GAL4>rpr*) or the two genetic controls (*dilp3GAL4* or *UAS-rpr* alone) on days 7, 14, 21, 28 and 42, on the food containing a healthy amount of sucrose (1×) or excess sucrose (8×). Statistical analysis of the data is shown in table 4. (*b*) Boxplots showing the levels of TAG measured in individual female flies with mNSC ablation (*dilp3GAL4>rpr*) or the two genetic controls (*dilp3GAL4* or *UAS-rpr* alone) that were wild-type or deleted for *dfoxo*, on days 7, 14 and 21 on the food containing excess sucrose (8×). The same colour code is used as in (*a*) and (*b*), and is given in (*a*). Statistical analysis of the data is shown in table 5. (*c*) Lifespans of female flies with mNSC ablation (*dilp3GAL4>rpr*) or the two genetic controls (*dilp3GAL4* or *UAS-rpr* alone) that were wild-type or deleted for *dfoxo* on the food containing excess sucrose (8×). Statistical analysis of the data is shown in table 6.

Mixed effects linear model analysis was performed using *nlme* package in R (http://CRAN.R-project.org/package=nlme) with measurement batch as the random effect. The effects of transgenes were again examined using two *a priori* contrasts: (i) ablation *versus* the controls, and (ii) *dilp3GAL4* control *versus UAS-rpr* control. When looking at the TAG content in the ablation on two sugar concentrations (figure 3*a* and table 4), the initial model included ‘age’, ‘transgene’ and ‘8× sugar’ as covariates, all of their pairwise interactions and the three-way interaction, and was simplified by sequentially removing non-significant interaction terms and covariates to give the final model. When looking at TAG in the ablation in the wild-type and *dfoxo* null backgrounds (figure 3*b* and table 5), the initial model included ‘age’, ‘transgene’ and ‘*dfoxoΔ*’ as covariates, all of their pairwise interactions and the three-way interaction, and was simplified by sequentially removing non-significant interaction terms.

**Table 4.** Statistical analysis of data presented in figure 3*a*. Mixed effects linear model with 227 observations (*n* ∼ 8) and assay batch as random effect. Note that the highest and lowest observations for each condition/time point were removed prior to analysis to guard against outliers. The effects of transgenes present were assessed using two *a priori* contrasts: (i) ablation (*dilp3GAL4>rpr*) *versus dilpr3GAL4* and *UAS-rpr* alone controls, and (ii) *dilp3GAL4* control *versus UAS-rpr* control. Food was modelled as a categorical variable with 1× sugar as reference. Colon (‘:’) indicates interaction term. The initial model included ‘age’, ‘transgene’ and ‘8× sugar’ as covariates, and all of their interactions, and was subsequently simplified by sequentially removing non-significant terms. The coefficient estimates are expressed in µmol TAG per fly.

coefficient	estimate	s.e.	*t*	*p*-value
intercept	0.18	0.010	18	<10^−4^
transgene				
ablation *versus* controls	−2.9 × 10^−3^	3.2 × 10^−3^	−0.90	0.37
* dilp3GAL3 versus UAS-rpr*	0.023	5.6 × 10^−3^	4.1	10^−4^
8× sugar	0.037	6.5 × 10^−3^	5.7	<10^−4^
8× sugar : transgene				
ablation *versus* controls	−0.020	4.6 × 10^−3^	−4.3	<10^−4^
* dilp3GAL3 versus UAS-rpr*	2.2 × 10^−5^	8.1 × 10^−3^	2.7 × 10^−3^	1.0

**Table 5.** Statistical analysis of data presented in figure 3*b*. Mixed effects linear model with 136 observations (*n* ∼ 8) and assay batch as random effect. Note that the highest and lowest observations for each condition/time point were removed prior to analysis to protect against outliers. The effects of transgenes present were assessed using two *a priori* contrasts: (i) ablation (*dilp3GAL4>rpr*) *versus dilpr3GAL4* and *UAS-rpr* alone controls, and (ii) *dilp3GAL4* control *versus UAS-rpr* control. Food was modelled as a categorical variable with 1× sugar as reference. The initial model included ‘age’, ‘*dfoxoΔ*’ and ‘8× sugar’ as covariates, and all of their interactions and was subsequently simplified by sequentially removing non-significant terms. The coefficient estimates are expressed in µmol TAG per fly.

coefficient	estimate	s.e.	*t*	*p*-value
intercept	0.18	0.032	5.4	< 10^−4^
age (day)	0.007	1.1 × 10^−3^	6.3	< 10^−4^
transgene				
ablation *versus* controls	−0.019	4.4 × 10^−3^	−4.3	< 10^−4^
* dilp3GAL3 versus UAS-rpr*	0.010	0.008	1.3	0.19
*dfoxo*Δ	0.029	0.012	2.3	0.023

In all cases, *p* = 0.05 was used as the significance cut-off.

## 3. Results

### (a) Ablation of median neurosecretory cells cannot overcome the detrimental effects of excess sugar on fly lifespan

We first examined whether the ablation of mNSCs can alter the response of lifespan to excess dietary sugar. The ablation of the mNSCs was achieved by combining a construct encoding the pro-apoptotic gene *reaper* (*rpr*) under the control of a GAL4-responsive Upstream Activating Sequence (*UAS-rpr*) with the *dilp3GAL4* driver that restricts the expression of the GAL4 TF to the mNSCs, as described previously [19]. The lifespan of the ablated flies (*dilp3GAL4>rpr*) and the two genetic controls (*dilp3GAL4* or *UAS-rpr* alone) were measured on food containing the optimal sugar concentration (5% w/v sucrose, referred to as 1× sugar) [8] or an 8× excess of sugar (figure 1*a*). Note that all experiments were performed on mated females.

As previously reported [7], excess sugar substantially reduced the lifespan of all genotypes, shortening median lifespan by an average of 43% (pairwise comparison of survival under the two sugar concentrations by Logrank, *p* < 10^−3^ for all genotypes, figure 1*a*). mNSC ablation extended median lifespan under both 1× and 8× sugar conditions by 21% and 28%, respectively (pairwise comparison of survival in the ablation to each control under both conditions by Logrank, *p* < 10^−3^, figure 1*a*).

Pairwise comparisons are not sufficient to test whether the excess sugar had a differential effect on the lifespan of the mNSC ablation. To do this, we performed Cox Proportional Hazards (CPH) analysis. This type of analysis can look at the effects of different covariates and their interaction(s) [21]. To test the effects of mNSC ablation in this and all other analyses, we employed an *a priori* contrast comparing the effect of the ablation against both controls. A second contrast was employed to test for differences between the two controls.

Using CPH analysis, we found that, while the effect of the ablation and high sugar were both highly significant (*p* < 2 × 10^−16^, table 1), there was no significant interaction between the treatments. Hence, even though the ablation of the mNSCs can extend fly lifespan in the presence of high sugar, relative to the two genetic controls observed under the same conditions, it cannot specifically remedy the detrimental effects of excess sugar.

Absence of a significant interaction between mNSC ablation and the high-sugar diet also indicated that the detrimental effects of excess sugar are not mediated by changes to IIS signalling. To further investigate this, we examined whether excess sugar can reduce the lifespan of flies mutated for the TF that is the key effector of IIS for lifespan, *dfoxo* [19]. We found that excess sugar equally reduced the lifespan of *dfoxo* null and wild-type flies (figure 1*b*). Again, CPH analysis identified very strong, significant individual effects of *dfoxo* deletion and high sugar (*p* < 2 × 10^−16^, table 2), but found no evidence for a significant interaction between the two treatments. Therefore, in the fly, the detrimental effects of sugar are independent of both the upstream and downstream components of IIS, and hence not caused by changes to IIS.

### (b) Median neurosecretory cell ablation improves age-related neuromuscular decline irrespective of sugar content

To further examine the physiological interaction between high dietary sugar and the beneficial effects of reduced IIS, we examined the performance of the fly neuromuscular system during ageing. We scored the ability of flies to climb a vertical surface because this is a suitable, age-sensitive measure of the functioning of this organ system [22]. We examined the climbing ability of the ablated flies and the control genotypes on both the 1× and 8× sugar food, by scoring the number of poor climbers (defined as climbing below 4 cm in our assay), medium climbers (above 4 cm and below 22 cm) and high climbers (above 22 cm) during the course of their lifespan (figure 2). Note that after some 40 days no flies remained alive on 8× sugar food, and hence we could not monitor their climbing ability further.

The data thus obtained are counts of flies belonging to ordered categories of low, medium and high climbers. The data are plotted in figure 2 as cumulative proportions of medium and high climbers (climbing above 4 cm) and high climbers (climbing above 22 cm). We analysed the data directly, rather than deriving a ‘performance index’ as has been done by others [20], using a mixed effects ordinal logistic model (table 3). We found that the climbing ability of all flies significantly decreased with age (*p* < 2 × 10^−16^, table 3). The ablated flies climbed better overall (*p* = 9.7 × 10^−5^, table 3) and for longer (age by ablation *versus* controls interaction, *p* = 1.9 × 10^−3^, table 3) compared to the genetic controls, indicating that a reduction in IIS can preserve the health of the neuromuscular system against ageing. Similar conclusions were reached when analysing the performance index (electronic supplementary material, figure S1 and table S1).

Interestingly, we found that there was a difference between the effects of high sugar on lifespan (figure 1*a*) and climbing ability (figure 2): the flies on 8× sugar were climbing as well, if not better, as the flies on 1× sugar even though their lifespans were substantially shorter. Indeed, increased sugar significantly counteracted the negative effect of age on climbing (age by sugar interaction, *p* = 0.012, table 3). This differential effect on climbing *versus* survival was particularly striking when the climbing data were plotted against the proportion dead: at the same proportion surviving/dead, the flies fed high sugar were better climbers (electronic supplementary material, figure S2). Hence, excess sugar does not affect equally all aspects of fly health and appears relatively beneficial for neuromuscular performance.

At the same time, the difference between the ablated and control flies was decreased by dietary sugar (sugar by ablation *versus* controls interaction, *p* = 1.1 × 10^−4^, table 3), indicating that high sugar diminishes the relative beneficial effects of the mNSC ablation on the performance of the neuromuscular system. This appears to be mostly owing to an improvement in the climbing ability of one control genotype on 8× food (figure 2 and table 3), and not owing to a decrease in the climbing ability of the ablated flies. Still, the ability of the ablated flies to climb well for longer (age by ablation *versus* controls interaction) was not significantly modified by high sugar, indicating that the ablation retains some effects on climbing irrespective of sugar content.

### (c) Median neurosecretory cell ablation can prevent fat accumulation on high-sugar diet independently of *dfoxo*

In adult flies, like in humans, excess dietary sugar causes increased accumulation of storage lipids in the form of TAG [7]. We next examined the effects of mNSC ablation on this diet-induced obesity phenotype by determining the TAG content of individual flies in the first 40 days of life. Compared to 1× sugar food, feeding the control genotypes with 8× sugar resulted in an increase in their TAG levels (figure 3*a*). At the same time, the flies with ablated mNSCs maintained similar levels of TAG on 1× and 8× sugar food, appearing resistant to this obesity (figure 3*a*). To test the significance of this effect, we analysed the data with a mixed effect linear model (table 4). The obesity response of the ablated flies to high-sugar feeding was significantly different from the response of the controls (sugar by ablation *versus* controls interaction *p* < 10^−4^, table 4), confirming that the ablation specifically blocked the effect of excess sugar on TAG accumulation.

The extension of lifespan caused by dampened IIS signalling is strictly dependent on the *dfoxo* transcription factor under optimal dietary conditions [19]. We next wanted to examine whether the ability of the ablated flies to resist excess-sugar-induced obesity also required *dfoxo*. We generated the ablated and control flies in wild-type and *dfoxo* null backgrounds and exposed them to 8× sugar food for three weeks, monitoring their TAG content (figure 3*b*). Analysing the data with a mixed effects linear model revealed that *dfoxo* deletion caused a slight but significant increase in TAG levels overall (*p* = 0.023, table 5), but it did not block the ability of the ablation to reduce the TAG levels on 8× food: the effect of the ablation remained significant (*p* < 10^−4^, table 5), and there was no evidence for a significant interaction with the effect of *dfoxo* deletion.

To determine whether there was a difference in the requirement for *dfoxo* between the effects of the ablation on high-sugar-induced obesity and high-sugar-induced mortality, we determined the lifespans of the flies combining the ablation of the mNSCs and *dfoxo* deletion (figure 3*c*). We found that the survival of *dfoxo* null flies could not be extended by the ablation of the mNSC on 8× food (figure 3*c*). CPH analysis revealed a significant interaction between the effects of *dfoxo* and the ablation for lifespan (*p* = 0.015, table 6), confirming that *dfoxo* is required for lifespan extension by IIS reduction on 8× sugar food. Hence, *dfoxo* is necessary for lifespan extension but not the resistance to obesity caused by the ablation of mNSCs in the presence of excess sugar.

**Table 6.** Statistical analysis of data presented in figure 3*c*. CPH model with 639 dead and 73 censored events. The effects of transgenes present were assessed using two *a priori* contrasts: (i) ablation (*dilp3GAL4>rpr*) *versus dilpr3GAL4* and *UAS-rpr* alone controls, and (ii) *dilp3GAL4* control *versus UAS-rpr* control. Colon (‘:’) indicates interaction term. The coefficient estimate is the natural log of the hazard ratio where a negative value indicates a beneficial effect on survival.

coefficient	estimate	s.e.	*z*	*p*-value
transgene				
ablation *versus* controls	−0.33	0.043	−7.7	1.7 ×10^−14^
* dilp3GAL3 versus UAS-rpr*	0.066	0.069	0.96	0.34
*dfoxo*Δ	2.8	0.12	22	<2 ×10^−16^
*dfoxo*Δ : transgene				
ablation *versus* controls	0.15	0.060	2.4	0.015
* dilp3GAL3 versus UAS-rpr*	−0.41	0.098	−4.2	3.0 ×10^−5^

## 4. Discussion

Our study shows that although the beneficial effects of IIS reduction are present irrespective of the sugar content of the food, IIS reduction cannot specifically combat the lifespan-shortening effects of a diet containing excess sugar. Furthermore, this reveals that the detrimental effects of sugar are not likely to be mediated by changes in IIS. Note that the IIS manipulation we employed alters the levels of the extracellular ligands, and different effects may be achieved by manipulations that directly alter intracellular IIS.

Our observations are somewhat different from those seen in *Caenorhabditis elegans*. In the nematode, addition of glucose to the diet completely suppresses the lifespan benefits of reduced IIS [23]. This is thought to result from glucose strongly inhibiting the activity of DAF-16, the FoxO-family TF that is strictly required for IIS-mediated lifespan extension [23,24]. Consistent with this, glucose does not further reduce the lifespan of *daf-16* mutant worms [23], while our study shows that excess sucrose further reduces the lifespan of *dfoxo* null flies. Despite the differences, in both organisms the reduction in IIS is unable to combat the detrimental effect of high sugar on lifespan. A recent report that a diet high in sugar reduces the beneficial effects of a mutant in the insulin receptor substrate, *chico*, which is otherwise long-lived [11], further substantiates this. How excess dietary sugar interacts with IIS to determine mammalian lifespan is unknown. However, based on observations in model organisms, it is likely that additional interventions will be required to achieve full anti-ageing benefits of treatments based on IIS reduction in populations that consume excessive amounts of sugar in their diet. This is potentially highly relevant for humans, whose diet is increasingly abundant in added sugar [2].

Surprisingly we found a contrast between the effects of high dietary sugar content on lifespan and climbing in *Drosophila*. While clearly detrimental for survival, excess sugar in the diet had a relatively beneficial effect on climbing ability. This was most clearly observed when comparing the proportion of the population dead to the proportion of good climbers at the same time point. The simplest explanation for this relative beneficial effect of high sugar on climbing ability is that excess sugar combats some metabolic or energy defect that occurs with age, and which, in part, results in the decline in climbing ability. Indeed, loss of energy stores has been observed in older flies [7]. At the same time, the relative beneficial effect of the ablation on climbing was reduced on high sugar, indicating that the ablation and high sugar act, in part, via the same mechanism to improve climbing ability. Hence, reduction in IIS may improve climbing ability, in part, by combating an age-dependent metabolic defect.

We also found that IIS reduction can prevent sugar-induced obesity in flies. This is consistent with the role of insulin in sugar uptake and conversion into fat in mammals [16]. Interestingly, this prevention of obesity is separable from changes in lifespan, indicating that obesity does not necessarily cause increased mortality in our experimental system. Similar observations have been made in humans, where 20% of obese individuals have normal metabolism and life expectancy [2]. Furthermore, a recent study indicates that the development of obesity may actually be protective against the effects of high-sugar diet in flies [25].

In flies, this anti-obesity effect of decreased IIS appears independent of *dfoxo*. Interestingly, it has recently been appreciated that, *in vivo*, insulin can regulate metabolic functions in the mouse liver in the absence of FoxO1 and its upstream insulin-responsive kinases Akt1 and Akt2 [26]. This indicates that a second, insulin-activated pathway can regulate metabolism in parallel to the canonical Akt–FoxO pathway. In *Drosophila*, several adult phenotypes are regulated by IIS independently of *dfoxo* [19], and the transcriptional response to IIS in this organism clearly involves other TFs [27], confirming the existence of *dfoxo*-independent pathways responding to IIS. On the other hand, mNSCs may be producing endocrine factors other than *dilp*s to regulate lipid storage.

While lifestyle and behavioural changes may be the preferred way to treat human obesity, they are not always sufficient, nor economically the most viable option [28]. Our results suggest that this *dfoxo*-independent pathway may be a suitable target for pharmacological or other treatments of obesity and warrants further investigation.

## 5. Conclusion

In this study, we examined the interaction between a diet containing unhealthy levels of sugar and a health-promoting reduction in IIS in the adult female fruit fly. We found that the reduction in IIS has beneficial effects on lifespan and ageing of the neuromuscular system but cannot specifically cure the lifespan-shortening effect of excess sugar. On the other hand, the reduction in IIS can block sugar-induced obesity in the adult fly, independently of *dfoxo*. These findings have the potential to inform future studies aimed at uncovering interventions into human ageing, age-related diseases and obesity.

## Supplementary Material

Supplementary Materials

## Data accessibility

All data are deposited in Dryad (manuscript Dryad ID: doi:10.5061/dryad.t04v4).
