# Supplementary material for: Ablation of insulin-producing cells prevents obesity but not premature mortality caused by a high-sugar diet in Drosophila
Source: Proc Biol Sci. 2015 Feb 7;282(1800):20141720. doi: 10.1098/rspb.2014.1720 (PMC4298201; doi:10.1098/rspb.2014.1720)
Supplement: Supplementary Materials [file rspb20141720supp1.pdf]

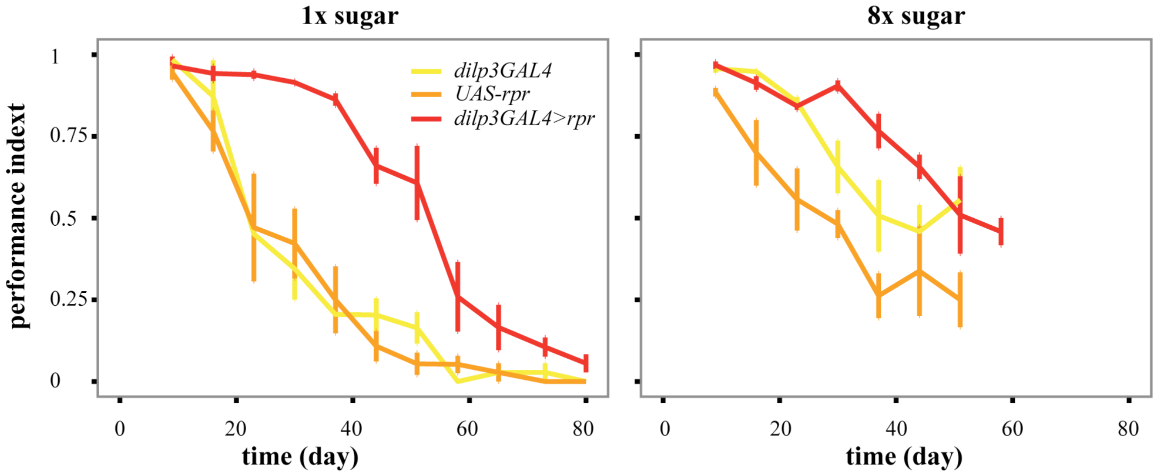

Supplemental Figure 1

The climbing data, presented in Figure 2, were used to calculate the performance index as ½(total number of flies observed + number of high climbers - number of low climbers)/total number of flies observed). The mean performance index and the standard error of the three cohorts are plotted. Statistical analysis of the data is given in Supplemental Table 1.

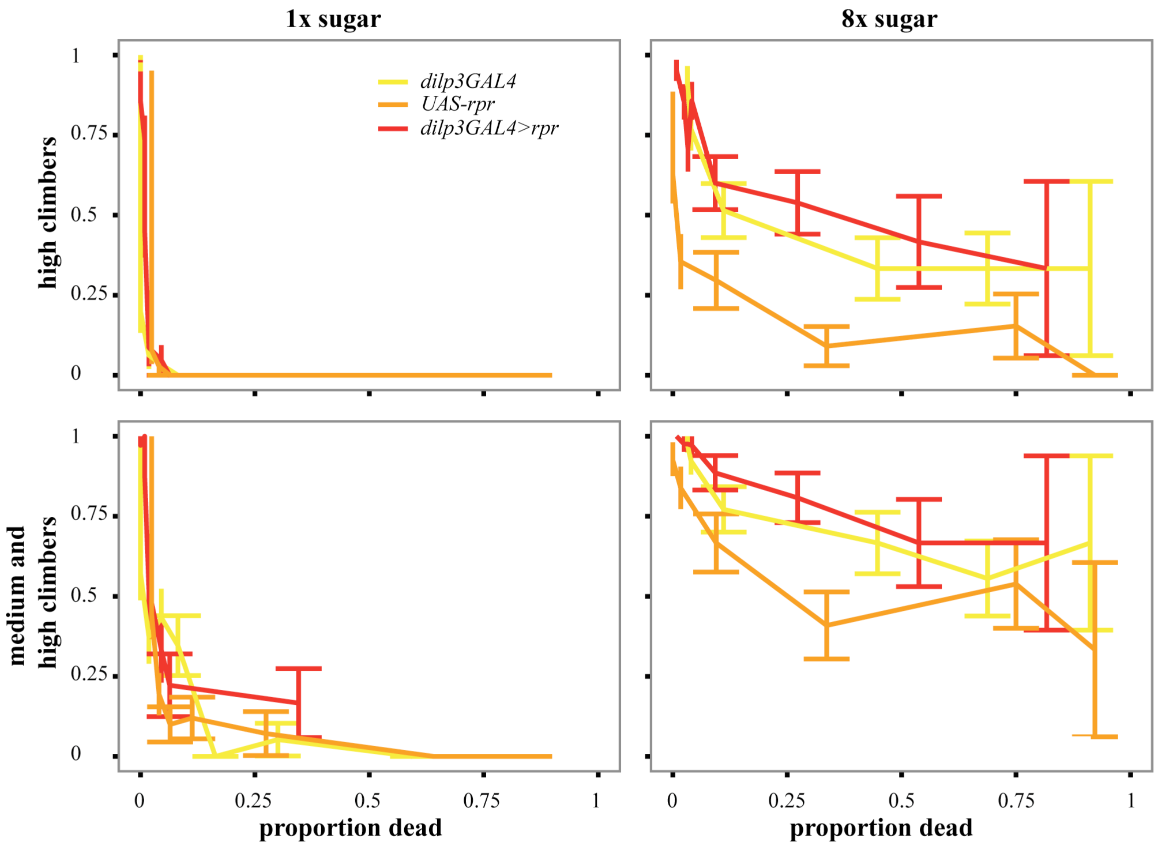

Supplemental Figure 2

The climbing and survival data presented in Figures 1a and 2 were combined to show the relationship between the proportion high climbers (top) or medium and high climbers (bottom panels) and the proportion of flies dead at the same time point, for both 1x (left) and 8x sugar (right panels). Standard errors are plotted for the climbing data.

Supplemental Table 1 Statistical analysis of data in Supplemental Figure 1
Linear model (n=3). The effects of transgenes present were assessed using two *a priory* contrasts: (1) ablation (*dilp3GAL4>rpr*) *versus dilpr3GAL4* and *UAS-rpr* alone controls, (2) *dilp3GAL4* control *versus UAS-rpr* control. Food was modelled as a categorical variable with 1x sugar as reference. Column (":") indicates interaction term. The initial model included "age", "transgene" and "8x sugar" as covariates, and all of their interactions and was subsequently simplified by sequentially removing non-significant terms. The coefficient estimates have no units since they are derived from the performance index, which also has no units; a positive value indicates an improvement in climbing ability

Coefficient	Estimate	SE	t	p
**intercept**	0.99	0.030	33	$<2\times10^{-16}$
**age** (day)	-0.013	5.9×10^{-4}	-23	$<2\times10^{-16}$
**8x sugar**	0.085	0.025	3.4	8.1×10^{-4}
**transgene**				
ablation *versus* controls	0.10	0.011	9.5	$<2\times10^{-16}$
dilp3GAL3 versus UAS-rpr	8.3×10^{-3}	0.018	0.46	0.64
**8x sugar : transgene**				
ablation *versus* controls	-0.034	0.016	-2.0	0.043
dilp3GAL3 versus UAS-rpr	0.097	0.029	3.3	1.1×10^{-3}
